# A study of temperature variability on admissions and deaths for cardiovascular diseases in Northwestern China

**DOI:** 10.1186/s12889-023-16650-3

**Published:** 2023-09-08

**Authors:** Shan Zheng, Xiaofei Zhang, Wenzhi Zhu, Yonghong Nie, Ximeng Ke, Shaodong Liu, Xue Wang, Jinlong You, Feng Kang, Yana Bai, Minzhen Wang

**Affiliations:** 1 Institute of Epidemiology and Statistics, School of Public Health, Lanzhou University, Lanzhou, 730000 China; 2 Center for Immunological and Metabolic Diseases (CIMD), MED-X Institute, the First Affiliated Hospital of Xi’an Jiaotong University, Xi’an, China; 3 Jinchang Center for Disease Control and Prevention, Jinchang, 737100 China; 4 Workers’ Hospital of Jinchuan Group Co., Ltd, Jinchang, 737103 China

**Keywords:** Temperature variability, Admissions and deaths, Cardiovascular diseases, Distributed lag nonlinear model

## Abstract

**Objective:**

To explore the effect of temperature variability (TV) on admissions and deaths for cardiovascular diseases (CVDs).

**Method:**

The admissions data of CVDs were collected in 4 general hospitals in Jinchang City, Gansu Province from 2013 to 2016. The monitoring data of death for CVDs from 2013 to 2017 were collected through the Jinchang City Center for Disease Control and Prevention. Distributed lag nonlinear model (DLNM) was combined to analyze the effects of TV (daily temperature variability (DTV) and hourly temperature variability (HTV)) on the admissions and deaths for CVDs after adjusting confounding effects. Stratified analysis was conducted by age and gender. Then the attribution risk of TV was evaluated.

**Results:**

There was a broadly linear correlation between TV and the admissions and deaths for CVDs, but only the association between TV and outpatient and emergency room (O&ER) visits for CVDs have statistically significant. DTV and HTV have similar lag effect. Every 1 ℃ increase in DTV and HTV was associated with a 3.61% (95% CI: 1.19% ~ 6.08%), 3.03% (95% CI: 0.27% ~ 5.86%) increase in O&ER visits for CVDs, respectively. There were 22.75% and 14.15% O&ER visits for CVDs can attribute to DTV and HTV exposure during 2013–2016. Males and the elderly may be more sensitive to the changes of TV. Greater effect of TV was observed in non-heating season than in heating season.

**Conclusion:**

TV was an independent risk factor for the increase of O&ER visits for CVDs, suggesting effective guidance such as strengthening the timely prevention for vulnerable groups before or after exposure, which has important implications for risk management of CVDs.

**Supplementary Information:**

The online version contains supplementary material available at 10.1186/s12889-023-16650-3.

## Introduction

The Global burden of diseases (GBD) study [1] found that the prevalence of cardiovascular diseases (CVDs) almost doubled from 1990 to 2019, from 271 million in 1990 to 523 million in 2019, and the number of CVDs deaths increased steadily from 12.1 million to 18.6 million. CVDs has become the leading cause of death in the world [2–4], and it is also the main cause of premature death and rising the medical costs [1, 5, 6]. At least 3/4 of CVDs deaths in the world occurred in low-and middle-income countries, of which China has the largest number of CVDs deaths [7]. So far, a large number of epidemiological studies have reported the relationship between temperature changes and the occurrence and death of CVDs. Extreme temperatures (such as heat wave and cold wave), diurnal temperature range (DTR) and temperature change between neighboring days were found to be associated with the occurrence of CVDs and increased risk of death [8–13].

Temperature variability (TV) is also an important meteorological index to reflect temperature changes. In recent years, some epidemiological studies have proposed that the effects of temperature changes on cardiovascular health can be evaluated by considering both intra-day and inter-day temperature differences. Guo et al. [14] proposed the daily temperature variability (DTV) firstly by calculating the standard deviation of the minimum and maximum temperature during the exposure days, and then evaluated its impact on the mortality based on the Multi-Country multi-City (MCC) collaborative network. Subsequently, a number of studies assessed the association between DTV and deaths from specific causes of disease, especially CVDs [15–17]. For example, Yang et al. [17] analyzed the effects of DTV on death in 31 cities in China and found that for every 1℃ increase in DTV, the number of CVDs deaths increased by 0.65% (95%CI: 0.24%, 1.05%). Ma et al. [18] analyzed the impact of DTV on deaths in 47 prefectures of Japan from 1972 to 2015, found that for every 1℃ increase in DTV, the number of deaths from cardiovascular disease and cerebrovascular diseases increased by 1.11% (95%CI: 1.01%, 1.22%) and 1.09% (95%CI: 0.94%, 1.24%), respectively. In addition to the DTV-death association, some studies have also analyzed the effect of DTV on the number of hospitalizations for CVDs. Zhao et al. [19] used a national data from Brazil to quantify the link between DTV and the number of admissions for hospitalizations and found the risk of hospitalization significantly associated with the increase of DTV over a 16-year period. When a 1 ℃ increase in DTV, the OR for arrhythmia was 1.012 (95%CI: 1.010 ~ 1.015) [20], when a 5℃ increase in DTV, the OR for ischemic heart disease was 1.019 (95%CI: 1.013 ~ 1.025) [21]. Tian et al. [22] conducted a study in 184 Chinese cities and found that for every 1 ℃ increase in DTV, the total number of admissions for CVDs, coronary heart disease, heart failure, arrhythmia and ischemic stroke increased by 0.44% (95%CI: 0.32% ~ 0.55%), 0.31% (95%CI: 0.20%-0.43%)、0.48% (95%CI: 0.01%-0.96%), 0.34% (95%CI: 0.01%-0.67%), 0.82% (95%CI: 0.59%-1.05%), respectively.

Besides, some studies suggested that hourly temperature variability (HTV) can be obtained by calculating the standard deviation of hourly temperature during the exposure day, arguing that HTV could theoretically better capture the temporal variation in temperature at a finer scale [23–25]. Two studies conducted by Hu et al. [26, 27] in Zhejiang Province of China and found that there was a significant correlation between HTV and all causes of death, which reported that for every 1 ℃ increase in HTV, the total number of deaths increased by 1.53% (95%CI: 1.31%, 1.73%), and the number of deaths for CVDs increased by 1.70% (95%CI: 1.30%, 2.10%).

The above studies were generally consistent in their findings, suggesting that increased DTV or HTV exposure might increase the risk of hospitalization and death from CVDs. However, few studies have been conducted in the effects of TV on the outpatient and emergency room (O&ER) visits form CVDs. In addition, although previous researches have used multi-cities data to reflect the impact of TV on a large scale, the indicators of TV involved in previous studies were relatively single, and comprehensive analyses focusing on the effects of both DTV and HTV on the number of O&ER visits, hospitalizations, and deaths form CVDs are rare. Therefore, based on the existing research foundation, this study evaluated the effects of the two temperature variation indicators (DTV and HTV) in the region on the number of hospital admissions and deaths of CVDs in the population comprehensively, so as to provide more comprehensive regional evidences for the effects of weather changes on CVDs.

## Material and methods

### Admissions and deaths Data for CVDs

Jinchang is a Northwestern city in China, which located at 101°04′35"- 102°43′40" east longitude and 37°47′10"- 39°00′30" north latitude. Jinchang belongs the inland arid region with the continental temperate arid climate, and has the large temperature difference between day and night and has four distinct seasons. The annual average temperature, maximum and minimum temperature were 9.2℃, 30.0℃ and -16.2℃, respectively. So the average diurnal temperature range was up to 25.3℃. It is an area with fragile natural ecological environment in western China, which has geographical advantages for studying the effects of temperature variability on population health.

The four largest general hospitals in Jinchang city were chosen to collect O&ER visits and hospitalization data (from 1 January 2013 to 31 December 2016), the admissions of the four hospitals can cover more than 90% admissions in the city. Due to the delay for the O&ER information registration of one hospital, only three hospitals' O&ER cases for CVDs were collected. The case information mainly included admission date, visits number, age, gender, admitting diagnosis, final discharge diagnosis and ICD-10 (International Classification of Diseases-10) code, etc. If the case records included regular drug withdrawals, duplicate visits number, missing information for diagnoses and unknown diagnoses, these cases were deleted. The monitoring data of all causes of death in Jinchang City from January 1, 2013 to December 31, 2017 were collected from the Jinchang City Center for Disease Control and Prevention. The death case records included gender, age, time of death, place of death, direct cause of death and its ICD-10 code, etc. All cardiovascular diseases, hypertension, coronary heart disease (CHD), and stroke were selected from the medical record and the cause of death database according to the ICD-10 code: I00-I99, I10-I15, I20-I25, I60-I65.

### Meteorological and air pollution data

Daily meteorological data from January 1, 2013 to December 31, 2017 were collected from Jinchang Meteorological Bureau, including daily minimum, mean and maximum temperature, hourly temperature (2:00, 8:00, 14:00, 20:00), relative humidity and mean wind speed. The air pollution data in the same period came from Jinchang Environmental Monitoring Station, mainly including PM_10_, SO_2_, and NO_2_. Spatial distribution of the environmental monitoring station and the hospitals in Jinchang was shown in the previous research [28]. This study used the proximity point mean values to supplement the missing data of meteorological pollutants to form a complete meteorological pollutant data series. Two composite indexes, which can account for both intra- and inter-day temperature variability, were used for exposure assessment of short-term temperature variability (TV) in this study. Consistent with previous researches, daily temperature variability (DTV) was generated from the standard deviations (SD) of several days' daily minimum and maximum temperatures [14, 25], whereas hourly temperature variability (HTV) was developed by calculating SDs of four hourly temperatures during the exposure days [23, 24, 29].

### Statistical methods

#### Time series model

This study is an ecological study, which will explore the effects of TV on the hospital admissions and deaths of CVDs. To avoid the influence of over-dispersion of Poisson distribution on effect estimation, quasi-Poisson regression model combined with distributed lag nonlinear model (DLNM) was used as our core model in this study [30]. The specific modeling process was as follows:

1. Firstly, a natural cubic spline (ns) of time with 7 degrees of freedom (df) per year was adjusted to control the long-term trend and seasonality [15, 22].
2. Holiday effect and Day of week (DOW) effect were adjusted in the model [22].
3. Two natural cubic spline function was used for daily mean temperature with 4 df and lag over time up to 21 days respectively to accommodate the nonlinear and lagged effects of ambient temperature [22, 31]. Furthermore, a ns of relative humidity (RH) with 3 df was also adjusted in the model.
4. In this study, TV was induced into the model in the linear term as previous studies have observed linear effect of TV [14, 22, 23], and then estimate the excess risk (ER) of CVDs for every 1℃ change of TV. We also assessed separately the effects of TV at various exposure days (from lag 01 days to lag 07 days) on admissions and deaths for CVDs to better understand the lag effect. For example, TV at lag 01 day (TV01) was derived by calculating the SD of temperature exposure on the same day and 1 day before, TV02 was the SD for the preceding 3 days’ exposure, and so on.

The specific model [30] is as follow:

$${\varvec{L}}{\varvec{o}}{\varvec{g}}\left[{\varvec{E}}\left({{\varvec{Y}}}_{{\varvec{t}}}\right)\right]=\boldsymbol{\alpha }+{\varvec{\beta}}\left({{\varvec{D}}{\varvec{T}}{\varvec{V}}}_{{\varvec{t}}}/{{\varvec{H}}{\varvec{T}}{\varvec{V}}}_{{\varvec{t}}}\right)+{{\varvec{\beta}}}_{1}{{\varvec{T}}{\varvec{e}}{\varvec{m}}{\varvec{p}}}_{{\varvec{t}},{\varvec{l}}}+{\varvec{n}}{\varvec{s}}\left({{\varvec{R}}{\varvec{H}}}_{{\varvec{t}}},{\varvec{d}}{\varvec{f}}\right)+{\varvec{n}}{\varvec{s}}\left({\varvec{t}}{\varvec{i}}{\varvec{m}}{\varvec{e}},{\varvec{d}}{\varvec{f}}\right)+{\varvec{\gamma}}{{\varvec{h}}{\varvec{o}}{\varvec{l}}{\varvec{i}}{\varvec{d}}{\varvec{a}}{\varvec{y}}}_{{\varvec{t}}}+{\varvec{\delta}}{{\varvec{D}}{\varvec{O}}{\varvec{W}}}_{{\varvec{t}}}$$

where t refers to the observation days; Y_t_ is the number of admissions or deaths for CVDs in t day; E(Y_t_) is the expected number of admissions or deaths for CVDs in t day; ns refers to natural cubic splines; *α* is the intercept of the model; *β* is the coefficient with 1 unit increase of TV (DTV or HTV); Temp_t,l_ is the two-dimensional cross-basis matrix produced by DLNM, l is the lag days. *γ* and *δ* are the coefficient with holiday and DOW, respectively.

- (5) A natural cubic spline with 3 df for TV was included in the model to fit the relationship between TV and admissions or deaths for CVDs [23]. The confounding factors adjusted in the model were same as the above. According to the minimum value of Akaike information criterion for quasi-Poisson (Q-AIC), the optimal lag days were selected for fitting [12, 18].

In order to fully explore the effects of TV on admissions or deaths for CVDs, the stratified analysis in different gender (male and female) and age (< 60 and ≧60 years old) were conducted. The statistical differences between groups was compared by the formula [32]: $\left({{\varvec{\beta}}}_{1}-{{\varvec{\beta}}}_{2}\right)\pm 1.96\times \sqrt{{{\varvec{S}}{\varvec{E}}}_{1}^{2}+{{\varvec{S}}{\varvec{E}}}_{2}^{2}}$ *β*_*1*_ and *β*_*2*_ are the estimates for the two groups, *SE*_*1*_ and *SE*_*1*_ are their respective standard errors.

Jinchang is a typical central heating city. Considering the large difference between indoor and outdoor temperature in heating season, the meteorological observation data cannot well reflect the actual exposure of human body, this study divided the whole year into heating season (November to next year March) and non-heating season (April to October) to further analyze the seasonal effect of TV on admissions and deaths for CVDs.

### AF and AN calculation

The attribution fraction (AF) is used to indicate the degree to which the risk is specifically attributed to exposure factors, which can be calculated by the relative risk (RR) of the exposure factors. The specific formula [33] is as follows:

$$\begin{array}{ccc}{\mathrm{AF}}_{\mathrm{t}}=\left(\mathrm{RRt}-1\right)/{\mathrm{RR}}_{\mathrm{t}}& {\mathrm{AN}}_{\mathrm{t}}={\mathrm{AF}}^{*}{\mathrm{N}}_{\mathrm{t}}& {\mathrm{RR}}_{\mathrm{t}}=\mathrm{exp}\left({\upbeta }_{\mathrm{t}}\right)\end{array}$$

where AN_t_ refers to the attributable number on day t, N_t_ refers to the number of admissions or deaths for CVDs, RR_t_ refers to the relative risk of exposure to TV on day t.

### Sensitivity analyses

In order to determine whether air pollutants will have an impact on the TV effect, the solid pollutants (PM_10_) and gaseous pollutants (SO_2_ and NO_2_) which have the same lag days as TV were included in the model by linear formal, to further clarify the confounding effect of air pollutants. Considering the potential nonlinear relationship between TV and diseases, this study also examined the exposure–response relationship by observing before and after the inflection point divided into two segments, respectively. In addition, the study further explored the effect of longer exposures of 10 and 15 days based on one week to verify the cumulative lag effect.

All the statistical analyses were performed with “DLNM” and “splines” packages in R software version 3.6.1. Two-sided statistical test was conducted, and effects of *P* < 0.05 were considered statistically significant.

## Results

### Data description

Table 1 showed daily admissions and deaths for CVDs in Jinchang from 2013 to 2016. The total number of O&ER visits, hospitalization and deaths was 138,367, 34,699 and 5474, respectively. And there were 95, 24 and 4 cases of O&ER visits, hospitalization and deaths for CVDs per day averagely, respectively.

**Table 1:** Distribution of daily admissions (2013–2016) and deaths (2013–2017) for CVDs in Jinchang

	N	Mean ± SD	Min	P_25_	P_50_	P_75_	Max
**O&ER visits**							
Total	138367	94.71 ± 58.23	3	54	94	119	347
Male	77798	53.25 ± 35.71	1	29	51	66	223
≥ 65 years	58249	39.87 ± 29.14	1	21	37	51	191
Hypertension	78229	53.54 ± 40.04	1	27	48	67	216
CHD	24175	16.55 ± 10.22	0	9	16	21	78
Stroke	8106	5.55 ± 4.04	0	3	5	8	28
**Hospitalization**							
Total	34699	23.75 ± 11.19	1	15	23	31	67
Male	18789	12.86 ± 6.55	1	8	12	17	49
≥ 65 years	18267	12.50 ± 6.02	0	8	12	16	36
Hypertension	9253	6.33 ± 4.35	0	3	6	9	39
CHD	8688	5.95 ± 3.19	0	4	6	8	19
Stroke	5310	3.63 ± 2.22	0	2	3	5	14
**Deaths**							
Total	5474	3.00 ± 1.92	0	2	3	4	18
Male	3082	1.69 ± 1.41	0	1	1	2	14
≥ 65 years	4259	2.33 ± 1.68	0	1	2	3	13
Hypertension	418	0.23 ± 0.50	0	0	0	0	4
CHD	1982	1.09 ± 1.11	0	0	1	2	7
Stroke	2085	1.14 ± 1.11	0	0	1	2	6

*Abbreviation N* Number, *SD* Standard deviation, *Px* xth percentiles, *Min*, Minimum, *Max*, maximum

During the study period (2013–2017), the average DTV was 6.73℃, which was higher than HTV (4.78℃). And the daily mean temperature and relative humidity were 9.95℃ and 40.18%, respectively. At the same period, the average concentrations of PM_10_, SO_2_ and NO_2_ were 104.17 μg/m^3^, 44.04 μg/m^3^ and 19.15 μg/m^3^, respectively (Table 2).

**Table 2:** Summary statistics of daily meteorological factors and air pollution concentrations in Jinchang (2013–2017)

	Mean ± SD	Min	P_25_	P_50_	P_75_	Max
Meteorological factors						
DTV(℃)	6.73 ± 2.07	0.90	5.25	6.85	8.25	12.65
In cold season	6.44 ± 1.94	1.40	5.15	6.52	7.75	11.75
In warm season	6.94 ± 2.13	0.90	5.45	7.15	8.65	12.65
HTV(℃)	4.78 ± 1.87	0.32	3.34	4.84	6.22	11.71
In cold season	4.78 ± 1.82	0.66	3.42	4.84	6.15	11.22
In warm season	4.77 ± 1.91	0.32	3.26	4.85	6.30	11.71
Mean temperature(℃)	9.95 ± 10.96	-16.40	0.18	11.00	19.30	30.70
Relative humidity(%)	40.18 ± 16.73	2.00	2700	39.00	50.00	96.00
Air pollution concentrations						
PM_10_(μg/m^3^)	104.17 ± 108.43	10.00	53.67	9.33	116.67	1783.33
SO_2_(μg/m^3^)	44.04 ± 37.93	2.00	17.33	34.33	58.67	281.00
NO_2_(μg/m^3^)	19.15 ± 8.76	3.00	12.67	18.00	24.67	62.00

*Abbreviation SD* Standard deviation, *Px* xth percentiles, *Min*, minimum, *Max*, maximum

### The effects of temperature variability on admissions and deaths for CVDs

**Fig. 1:**
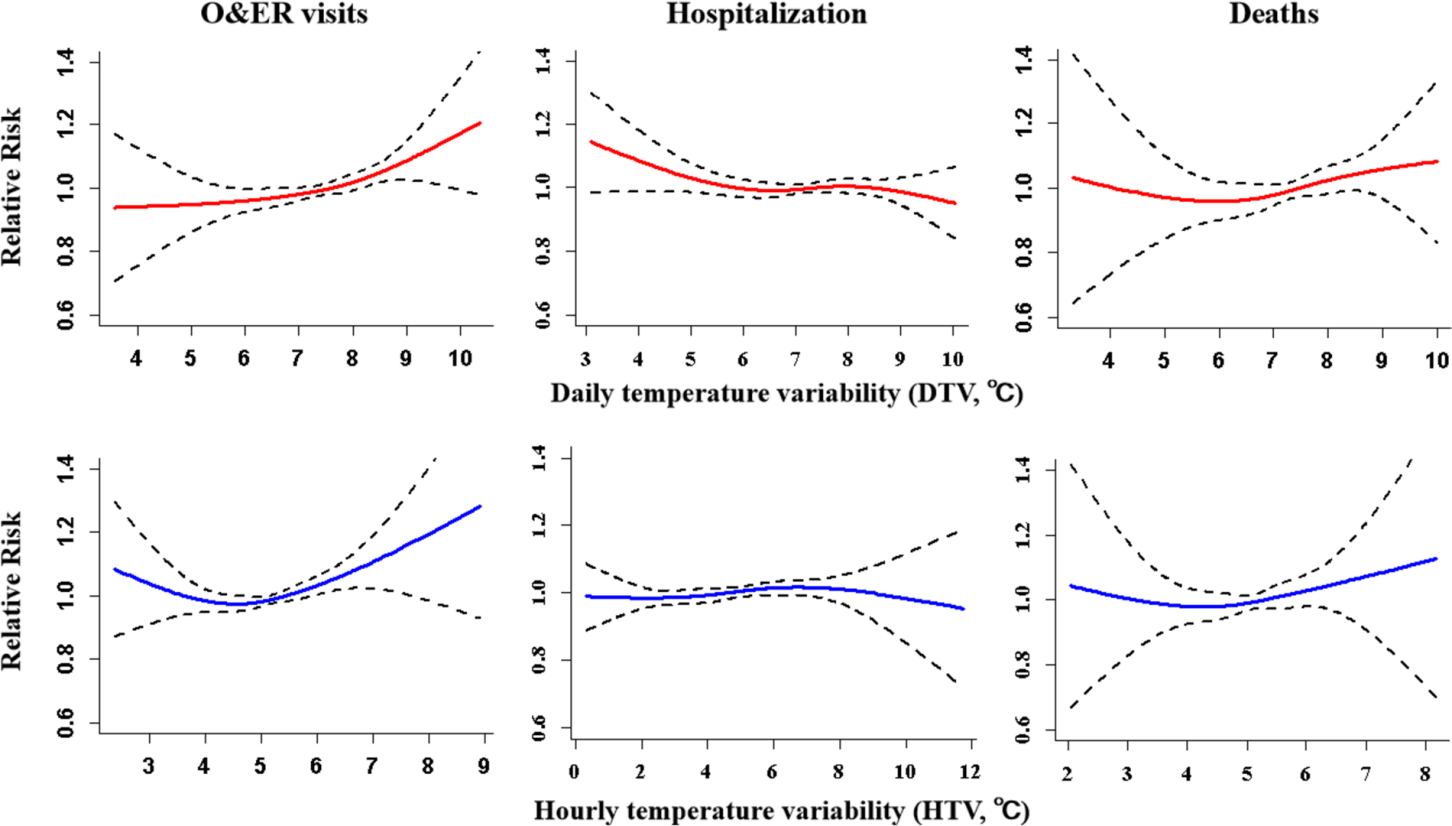
The exposure relationship between temperature variability and admissions and deaths for CVDs. Models were adjusted for time trend and seasonal effect, day of week, holiday, mean temperature and relative humidity. Based on Q-AIC, TV07, TV00, TV06 were selected for all O&ER visits, Hospitalization and Deaths

As shown in Fig. 1, there was a broadly linear association between TV and the admissions and deaths for CVDs. The total number of O&ER visits for CVDs showed an increasing trend with TV raised. However, the changes of hospitalization and deaths for CVDs showed no statistically significant with TV increases. The associations between TV and the admissions and deaths of CVDs for specific causes (hypertension, CHD, stroke) were similar with TV-CVDs (Figs. S1, S2, and S3).

**Fig. 2:**
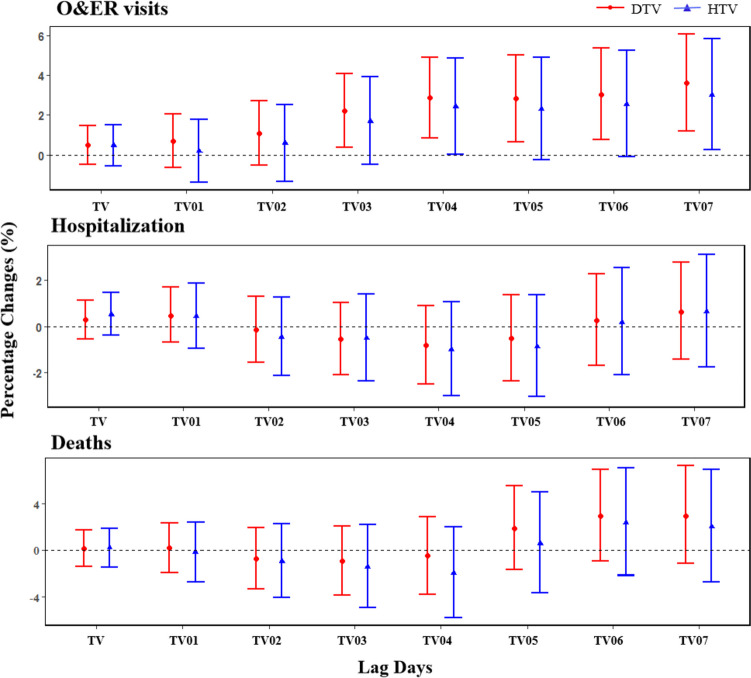
The percentage change with 95% CI in admissions and deaths for CVDs with 1℃ increase in TV at different exposure days. Models were adjusted for time trend and seasonal effect, day of week, holiday, mean temperature and relative humidity

Figure 2 shows the effects of TV on admissions and deaths for CVDs at different lag days. However, only the effects of TV on O&ER visits showed statistically significant. Generally, the lag effect models of DTV and HTV were basically the same, but the estimate values of DTV were slightly higher than HTV. The ER of O&ER visits for CVDs increased gradually with the increase of the cumulative exposure days of TV. For 1℃ increase in DTV and HTV at lag 07 day, the excess risks were 3.61% (95%CI: 1.19%, 6.08%) and 3.03% (95%CI: 0.27%, 5.86%).

The lag models of CVDs for specific causes were similar with that of CVDs (Figs. S4, S5, and S6). For 1℃ increase in DTV and HTV at lag 07 day, the ER of O&ER visits with hypertension were 4.31% (95%CI: 1.33%, 7.37%) and 4.10% (95%CI: 0.69%, 7.62%). For 1℃ increase in DTV and HTV at lag 04 day, the ER of O&ER visits for coronary heart disease were 3.75% (95%CI: 0.74%, 6.85%) and 2.64% (95%CI: -0.35%, 5.72%), and the ER of O&ER visits for stroke were 3.67% (95%CI: 0.41%, 7.04%) and 4.23% (95%CI: 0.37%, 8.24%), respectively. Based on above results, subsequent analyses were only for O&ER visits.

### Stratified analysis

**Fig. 3:**
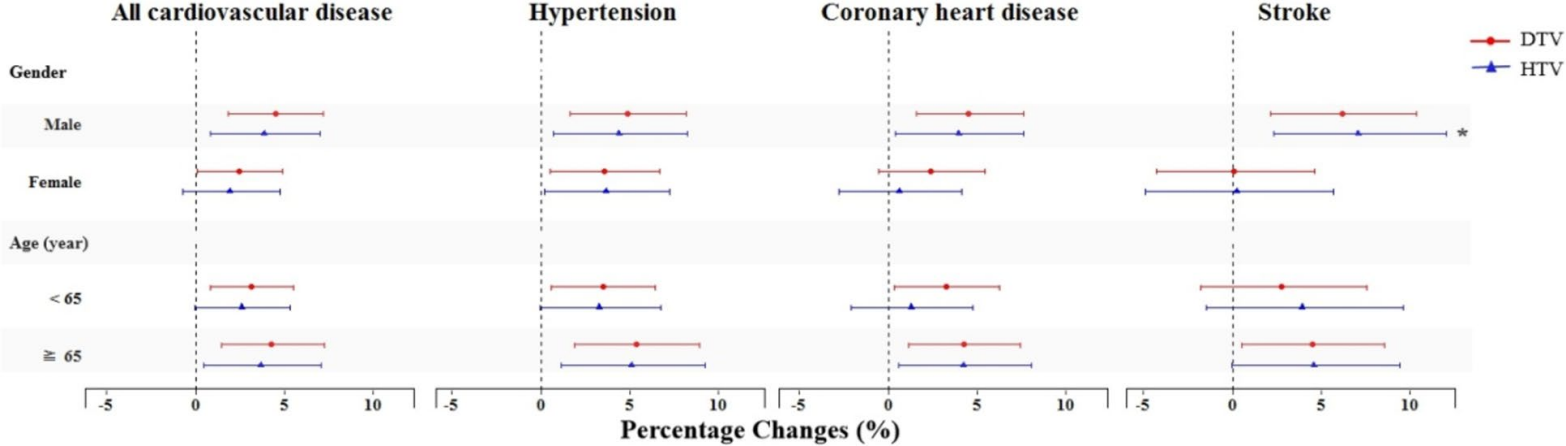
The percentage change with 95% CI in daily O&ER visits for CVDs with 1℃ increase in TV stratified by gender and age. Models were adjusted for time trend and seasonal effect, day of week, holiday, mean temperature and relative humidity. ^*^ indicates the statistically significant difference between the two groups

The lag days with the highest effect of DTV and HTV were selected for stratified analysis. As shown in Fig. 3, DTV and HTV have a greater impact on males with CVDs, especially for males with stroke (*P* < 0.05). For every 1℃ increase in DTV, the ER of O&ER visits for stroke in males and females were 6.20% (95%CI: 2.18%, 10.38%) and 0.11% (95% CI: -4.25%, 4.67%), respectively. For 1℃ increase in HTV, the ERs of stroke O&ER visits for males and females were 7.10% (95%CI: 2.33%, 12.09%) and 0.27% (95%CI: -4.87%, 5.69%). The estimate effects of TV among older people (≥ 65 years) were higher, although there was no significant difference between groups.

### Seasonal effect

**Fig. 4:**
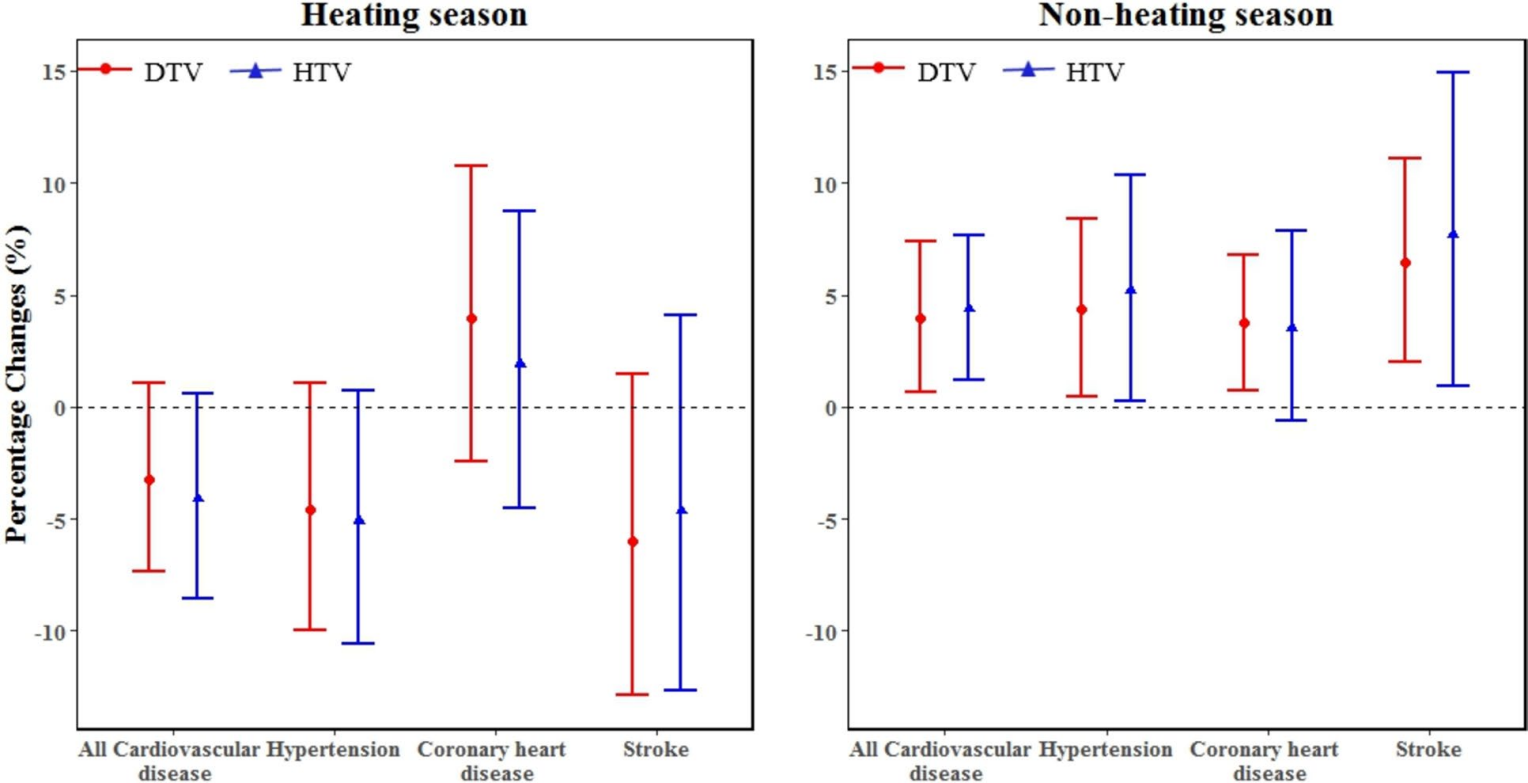
The percentage change with 95% CI in daily O&ER visits for CVDs with 1℃ increase in TV at different seasons. The maximum effects were observed at different exposure days, for Heating season, DTV05 and HTV05 were used for all cardiovascular diseases, DTV06 and HTV05 were used for hypertension, DTV07 and HTV06 were used for CHD, DTV05 and HTV06 were used for stroke; for Non-heating season, DTV07 and HTV04 were used for all cardiovascular diseases, DTV07and HTV07 were used for hypertension, DTV03 and HTV04 were used for coronary heart disease, DTV04 and HTV06 were used for stroke

Figure 4 shows the effects of TV on O&ER visits for CVDs in different season. The effects of TV in non-heating season were significantly higher than in heating season. There were no significant statistically differences in the effects of TV on O&ER visits for CVDs in heating season. In non-heating season, DTV increased by 1 ℃, the ERs of O&ER visits for all cardiovascular diseases, hypertension, CHD and stroke were 3.98% (95%CI: 0.69%, 7.37%), 4.37% (95%CI: 0.46%, 8.42%), 3.74% (95%CI: 0.76%, 6.82%), 6.45% (95%CI: 2.00%, 11.10%), respectively; HTV increased by 1 ℃, the ERs of O&ER visits for all cardiovascular diseases, hypertension, CHD and stroke were 4.37% (95%CI: 1.20%, 7.64%) and 5.19% (95%CI: 0.28%,10.34%), 3.54% (95%CI: -0.60%, 7.84%), 7.71% (95%CI: 0.94%, 14.94%), respectively.

### Sensitivity analysis

After further adjustment of air pollutants, the relationships between DTV, HTV and the number of O&ER visits for CVDs were consistent with the main model, but the estimate effects increased slightly (Table 3). After adjusting PM_10_, SO_2_ and NO_2_ in the model, with 1 ℃ increase in DTV, the ERs of O&ER visits for all cardiovascular diseases, hypertension, coronary heart disease and stroke were 3.78% (95%CI: 1.37%, 6.25%), 4.52% (95%CI: 1.56%, 7.58%), 3.70% (95%CI: 1.16%, 6.31%), 3.68% (95%CI: 0.42%, 7.04%); and for 1 ℃ increase in HTV, the ERs of O&ER visits for all cardiovascular diseases, hypertension, CHD and stroke were 3.35% (95%CI: 0.59%, 6.19%), 4.42% (95%CI: 1.02%, 7.94%), 2.68% (95%CI: -0.31%, 5.76%), 4.20% (95%CI: 0.34%, 8.20%), respectively.

**Table 3:** Results of the association between TV and O&ER visits for CVD after adjusted for air pollutants

	**DTV**		**HTV**	
	ER (%)	95% CI (%)	ER (%)	95% CI (%)
All cardiovascular disease				
Main model	**3.61**	**1.19 ~ 6.08**	**3.03**	**0.27 ~ 5.87**
Main model + PM_10_	**3.56**	**1.15 ~ 6.03**	**3.04**	**0.28 ~ 5.87**
Main model + SO_2_ + NO_2_	**3.82**	**1.40 ~ 6.30**	**3.26**	**0.50 ~ 6.10**
Main model + PM_10_ + SO_2_ + NO_2_	**3.78**	**1.37 ~ 6.25**	**3.35**	**0.59 ~ 6.19**
Hypertension				
Main model	**4.31**	**1.33 ~ 7.37**	**4.10**	**0.69 ~ 7.62**
Main model + PM_10_	**4.27**	**1.30 ~ 7.33**	**4.11**	**0.70 ~ 7.63**
Main model + SO_2_ + NO_2_	**4.56**	**1.58 ~ 7.62**	**4.32**	**0.91 ~ 7.84**
Main model + PM_10_ + SO_2_ + NO_2_	**4.52**	**1.56 ~ 7.58**	**4.42**	**1.02 ~ 7.94**
CHD				
Main model	**3.75**	**0.74 ~ 6.85**	2.64	-0.35 ~ 5.72
Main model + PM_10_	**3.70**	**1.16 ~ 6.31**	2.68	-0.31 ~ 5.76
Main model + SO_2_ + NO_2_	**3.70**	**1.16 ~ 6.31**	2.63	-0.36 ~ 5.72
Main model + PM_10_ + SO_2_ + NO_2_	**3.70**	**1.16 ~ 6.31**	2.68	-0.31 ~ 5.76
Stroke				
Main model	**3.67**	**0.41 ~ 7.04**	**4.23**	**0.37 ~ 8.24**
Main model + PM_10_	**3.65**	**0.39 ~ 7.01**	**4.13**	**0.28 ~ 8.13**
Main model + SO_2_ + NO_2_	**3.70**	**0.44 ~ 7.06**	**4.28**	**0.42 ~ 8.29**
Main model + PM_10_ + SO_2_ + NO_2_	**3.68**	**0.42 ~ 7.04**	**4.20**	**0.34 ~ 8.20**

Bold type indicate *P* < 0.05

Table S1 shows the exposure relationship between TV and admissions and deaths for CVDs with the two segments before and after the observed inflection point (DTV at 7℃ and HTV at 5℃). The results showed that there was no statistical significance at the lower TV before the inflection point, and it was evident that the linear measurement relationship of the higher TV may have a greater role in the overall trend (*P* < 0.05).

The percentage change with 95% CI in admissions and deaths for CVDs with 1℃ increase in TV at lag 10 and 15 days showed the general agreement with the above results, there was a statistically significant only for O&ER visits (Table S2). For 1℃ increase in DTV and HTV at lag 10 day, the excess risks were 4.47% (95%CI: 1.73%, 7.28%) and 3.80% (95%CI: 0.38%, 7.34%) for all cardiovascular diseases on O&ER visits. For lag 15 day, the excess risks were 5.10% (95%CI: 1.97%, 8.32%) and 4.95% (95%CI: 1.13%, 8.92%).

### Attributable burden of O&ER visits for cardiovascular diseases due to TV

Table 4 shows the attributable risk of O&ER visits for CVDs due to exposure to DTV and HTV. Both DTV and HTV could increase the number of O&ER visits for all cardiovascular diseases attribute to TV, and the attributable fraction of DTV (22.75%) was greater than HTV (14.15%). There were 31,437 O&ER visits for all cardiovascular diseases, which ccould attribute to DTV exposure during 2013–2016. Subgroup analysis showed that TV had a greater impact on male and people over 65 years old. For specific cause of CVDs, TV has the greatest attributable risk on the O&ER visits for hypertension, followed by CHD and stroke.

**Table 4:** Attributable fraction and number of temperature variability on O&ER visits for CVDs with each 1℃ increase

	**DTV**		**HTV**	
	AN (95%CI)	AF (%, 95%CI)	AN (95%CI)	AF (%, 95%CI)
All cardiovascular disease				
Total	**31473 (11498, 48252)**	**22.75 (8.31, 34.87)**	**19576 (2002, 34836)**	**14.15 (1.45, 25.18)**
Male	**21380 (9790, 30963)**	**27.48 (12.58, 39.80)**	**13744 (3310, 22685)**	**17.67 (4.25, 29.16)**
Female	**9876 (388, 17844)**	**16.31 (0.64, 29.46)**	5707 (-2351, 12711)	9.42 (-3.88, 20.99)
< 65	**16259 (4749, 25984)**	**20.29 (5.93, 32.43)**	9806 (-272, 18594)	12.24 (-0.34, 23.21)
≧65	**15385 (5762, 23216)**	**26.41 (9.89, 39.86)**	**9876 (1311, 17124)**	**16.95 (2.25, 29.40)**
Hypertension				
Total	**20692 (7178, 31594)**	**26.45 (9.18, 40.39)**	**14517 (27063, 24440)**	**18.56 (3.46, 31.24)**
Male	**12833 (4897, 19126)**	**29.27 (11.17, 43.63)**	**8693 (1450, 14671)**	**19.83 (3.31, 33.47)**
Female	**7778 (1378, 12918)**	**22.62 (4.01, 37.56)**	**5806 (367, 10358)**	**16.88 (1.07, 30.12)**
< 65	**9496 (1830, 15706)**	**22.17 (4.27, 36.67)**	6532 (-73, 12099)	15.25 (-0.17, 28.25)
≧65	**11215 (4552, 16416)**	**31.69 (12.86, 46.38)**	**7973 (2037, 12830)**	**22.53 (5.76, 36.25)**
CHD				
Total	**5690 (1273, 9243)**	**23.54 (5.26, 38.23)**	2929 (-423, 5812)	12.12 (-1.75, 24.04)
Male	**3914 (1510, 5858)**	**27.29 (10.53, 40.85)**	**2515 (311, 4364)**	**17.54 (2.17, 30.43)**
Female	1541 (-375, 3090)	15.67 (-3.81, 31.42)	306 (-1467, 1793)	3.11 (-14.92, 18.23)
< 65	**2662 (312, 4566)**	**20.49 (2.40, 35.16)**	803 (-1440, 2688)	6.18 (-11.09, 20.69)
≧65	**2892 (880, 4503)**	**25.86 (7.87, 40.25)**	**2092 (314, 3570)**	**18.70 (2.81, 31.92)**
Stroke				
Total	**1854 (240, 3130)**	**22.87 (2.95, 38.62)**	**1508 (151, 2627)**	**18.60 (1.86, 32.41)**
Male	**1682 (689, 2431)**	**35.09 (14.37, 50.70)**	**1387 (523, 2071)**	**28.92 (10.91, 43.20)**
Female	26 (-1220, 923)	0.79 (-36.83, 27.87)	44 (-938, 794)	1.33 (-28.32, 23.96)
< 65	665 (-514, 1508)	17.98 (-13.89, 40.77)	647 (-290, 1359)	17.49 (-7.85, 36.73)
≧65	**1194 (174, 1963)**	**27.09 (3.95, 44.55)**	880 (-8.26, 1586)	19.98 (-0.19, 35.98)

Bold type indicates *P* < 0.05

## Discussion

This study found that both DTV and HTV were risk factors for the increase in the number of O&ER visits for CVDs. However, the associations between TV and hospitalizations and deaths for CVDs were not found to be statistically significant. The results of subgroup analysis showed that male and the elderly may be more likely to be affected by TV. During the study period, the 22.75% and 14.15% of O&ER visits for all CVDs can be attributed to the exposure of DTV and HTV, respectively.

Previous studies reported linear correlation between TV and hospitalization and death [14, 16, 22, 23], this study also found the similar relationship. However, only the TV and O&ER visits’ association was the statistically significant, which may partly because the number of hospitalization and deaths for CVDs were not enough to show the effect of temperature change. Hospital factors like emergency services might be associated with the outcomes. Moreover, TV is the standard deviation of temperature, which is a finer index to evaluate temperature change. Compared with hospitalizations and deaths, O&ER visits are more quickly and timely ways for people to taken when expose to environmental factors and feel uncomfortable, so it could be better to explore the effect and lag effect between TV and clinical manifestations of CVDs.

This study showed that both DTV and HTV could significantly increase the number of O&ER visits for CVDs. The lag patterns of DTV and HTV were similar, but the estimate effects of DTV was slightly higher than HTV. For all cardiovascular diseases, with DTV and HTV increased by 1 ℃ at lag 07 day, the ER of O&ER visits for all cardiovascular diseases increased by 3.61% (95%CI: 1.19%, 6.08%) and 3.03% (95%CI: 0.27%, 5.86%), respectively. This result is consistent with some research results at home and abroad [16–18, 25–27]. For example, a study in Zhejiang, China [26] found that for every 1 ℃ increase in HTV07, the number of CVDs increased by 1.70% (95%CI: 1.30%, 2.10%); and a Japanese research [18] found that for every 1 ℃ increase in DTV07, the number of CVDs deaths increased by 1.11% (95%CI: 1.01%, 1.22%). Besides, a study [22] reported the association between DTV and hospital admission for CVDs reached maximum at lag 01 day with ER is 0.44% (95%CI: 0.32%, 0.55%), suggesting the lag effect of TV may be different with different disease outcomes.

Furthermore, this study analyzed the effect of TV on O&ER visits for hypertension, CHD and stroke. The results showed that the lag pattern of hypertension was consistent with that of all cardiovascular diseases, while that of CHD and stroke was the same. The estimate effect of TV on hypertension and stroke was significantly higher than that of CHD. Some studies have done related analysis for specific cause of CVDs and found similar results [12, 16, 18]. For example, a study in Hubei [16] found that for 1 ℃ increase in DTV, the number of deaths for stroke (1.72% (95%CI: 0.41% ~ 3.05%)) was higher than CHD (1.00% (95%CI: -0.84% ~ 2.88%)). The results of a Japanese study [18] also showed that for 1 ℃ increase in DTV, the number of deaths for cerebrovascular disease (1.09% (95%CI: 0.94% ~ 1.24%)) was a little higher than CHD (1.02% (95%CI: 0.78% ~ 1.26%)). There were also some studies found that TV had greater impact on the number of hospitalizations for stroke [20–22]. For example, Tian et al. [22] study showed for DTV increased by 1 ℃ at lag 01 day, the number of hospitalizations for CHD increased by 0.31% (95%CI: 0.20%-0.43%), less than ischemic stroke (0.82% (95%CI: 0.59%-1.05%)). Overall, both DTV and HTV can significantly increase the number of O&ER visits for CVDs, and have similar lag model. The estimated value of effects in this study was significantly higher than that in existing studies, which may be partly attributed to different disease outcomes. Besides, the differences in climate characteristics, social characteristics and population susceptibility between different study areas may also lead different results, more researches are needed in the future.

In this study, longer cumulative lags were found to show a higher risk of exposure, while the effects of same-day or short-term lags were not significant. This phenomenon has been seen in previous studies, such as in studies of daily temperature changes and heatwaves, which all found that higher risks of illness were obtained at longer cumulative lag days [12, 34, 35]. Firstly, in terms of the mechanism of the disease, the rapid change of temperature during one day will bring additional external environmental pressure to the body. If the temperature changes too much, especially when it exceeds the range of body regulation ability, it will cause the changes of corresponding physiology and biochemistry, previous studies have shown that the sudden changes of temperature was related to the increase of blood pressure, plasma cholesterol level, heart rate, platelet aggregation, peripheral vasoconstriction and plasma fibrinogen concentration [36–38]. These physiological changes may increase the burden on the human circulatory system, leading to the occurrence of cardiovascular diseases [39]. However, the body has its own immunity, as well as the ability to adapt to the external environment, and the occurrence of disease symptoms has a certain process. Transient changes in meteorological and environmental factors may be able to cause changes in the physiological level of the organism, but when it comes to causing obvious disease symptoms or more serious acute health events, the long-term cumulative exposure may be able to better explain this phenomenon. Secondly, the cumulative lagged effects of TV in this study were modeled as the mean of TV over a longer period of time, which mainly reflected the characteristics of TV over a period of time. From the perspective of a population epidemiology, this means that longer exposure in the population necessarily leads to a greater risk of disease. Moreover, a recent multi-country study reported that the highest estimates of temperature-variability–mortality appeared at different exposure days in different countries [14]. People living in hot areas were more sensitive to acute TV exposure than those in cold areas, whereas people living in moderate areas were more sensitive to long TV exposure than those in hot and cold areas. These differences may be caused by people adapting to their local climates via a range of physiological, behavioral, and technological adaptations [12, 40]. Therefore, it was plausible to hypothesize that the characteristics of study area, such as climates, geographical region, and population susceptibility, might also modify the lag pattern [22]. This still needs to be verified by more population-based studies in different regions.

In addition, some studies found that age, gender and other factors can modify the relationship between TV and the occurrence and deaths of CVDs. This study found that the estimate effects of TV on O&ER visits for CVDs in male and people over 65 years old were higher than that in women and people under 65 years old, especially in patients with stroke. That were similar to some of the existing studies. Zhang et al. [16] reported that older people are more likely to be affected by DTV- death than young people. Tian et al.’s study [22] showed that older people were more likely to be affected by DTV, but the effect values were basically the same between different genders. And Zhao et al. [19] also found DTV had a greater impact on hospitalization among men and the elderly. However, Zhao et al. [21] and Yang et al.’s studies [12] reported there was no significant difference in the effect of DTV on hospitalization and death among different gender and age groups, respectively. And Hu et al. [26] reported that women and the elderly are more sensitive to changes in HTV than men and young people. To sum up, in the effect of TV on CVDs, the effect modification of gender is not clear. When considering the modification effect of gender, the study of regional environment and population characteristics is a very important factor [41]. Two studies in western China showed that men were more likely than women to be engaged in diversified activities (agricultural and non-agricultural) [42, 43]. In western China, it may be still common for women to take care of their families and to be less exposed to the outside in the study area. With the changes of temperature, women can take corresponding measures in time to adapt to the temperature change, while men have more opportunities to work outside and may bear more pressure, which leads to a greater risk of exposure to TV, and usually cannot take corresponding measures to adapt to TV in time according to the changes of TV [38, 44]. The elderly as a susceptible population can be demonstrated in most studies, which may be related to the decline of physical function of the elderly. Generally speaking, the physiological function of the organs of the elderly is gradually weakening, especially the cardiopulmonary function, coupled with the high prevalence of chronic diseases in the elderly, so when the temperature changes greatly in a short period of time, the elderly may be more likely to have CVDs events [12, 45].

In some previous studies on the health effects of TV, it was found that there were differences in the effects of TV on disease occurrence or death in different season. For example, Guo et al. [14] found that there was a positive correlation between DTV and death in all seasons, but the estimated effect of DTV on death was the largest in the transitional season. The study of Zhang et al., [16] and Ma et al. [18] found that the correlation between DTV and death was stronger in the warm season than in the cold season. In addition, some studies have found that the effect of HTV on death has obvious seasonal variation, and the greatest effects were observed in warm season [24, 27]. The area of this study is located in the Northwestern China with a high altitude and cold weather. In winter, most of the work and life are completed indoors with indoor heating, which is less affected by the change of outdoor temperature, so the outdoor ambient temperature may not represent the actual personal exposure temperature well. This maybe partly explain why we observed the effects of TV on O&ER visits for CVDs in heating season were all have no statistical significant, suggesting indoor temperature should be considered in the future. And we also found that the effect of TV on O&ER visits for CVDs in the non-heating season was higher than heating season and whole year, consistent with the previous researches. At present, the reason for this phenomenon is not clear, which may be related to the differences of climate characteristics in different seasons, or it may be that high temperature events are more likely to occur in non-heating seasons, thus enhancing the effect of TV. In short, based on the previous studies, the results of this study suggest that future adaptation and intervention strategies to climate change should be aimed at not only heat waves, but also temperature variability, so as to reduce temperature-related health risks in hot season.

Attribution fraction is a more informative and policy-oriented measure than relative risk when describing the disease burden caused by a risk factor. This study found that 22.75% and 14.15% of the O&ER visits for all cardiovascular disease can be attributed to DTV and HTV, respectively. A previous study [20] found that 35,813 cases of arrhythmia hospitalization in Brazil during the study period could be attributed to exposure to DTV, accounting for 8.0% of arrhythmia hospitalization. However, little evidence on the attributable risk of TV was available, some studies reported the attributable risk of temperature. For example, two studies in China [46, 47] found that 17.1% and 14.5% of CVD and stroke deaths can be attributed to ambient temperature. By comparing with the results reported, our study found higher attributable risk of TV. This may be due to the stronger timeliness of O&ER visits, more studies are needed to convince this. Subgroup analysis found that male and elderly accounted for a higher proportion. This was consistent with previous reports that temperature attribution risk of CVD death increased with age [29, 46]. Therefore, the change of environmental temperature may indeed be a cause of the occurrence and death of CVD, but there are great differences due to the differences of geographical factor, climatic characteristic, urbanization level, socio-economic status and other factors. More research is still needed to further quantify the TV attribution risk of CVD and to provide more references for dealing with climate change in the future.

This study firstly analyzed the effects of TV on CVDs admissions and deaths through the three dimensions of O&ER visits, hospitalization and death. Some limitations also lay: (1) The study area belongs to the high altitude area of northwest China, and more studies on different climatic regions are needed to confirm the findings of this study, which will help to further explain the potential mechanism of TV as a risk factor for cardiovascular disease; (2) Since the population of Jinchang City is less than 500,000, the number of hospitalizations and deaths due to CVDs was less during the study period, it might be the insufficiency sample size for ecological study; (3) The data only from one meteorological station was used to calculate TV, which may lead to measurement errors.

## Conclusion

This study found that DTV and HTV were both independent risk factors for the increased risk of O&ER visits for CVD, and they have similar effects and lag patterns on CVDs. The results of subgroup analysis showed that men and the elderly may be sensitive groups, especially in stroke diseases. Therefore, this study suggested that in the context of climate change, public awareness and coping capacity of the impact of temperature variability on CVDs health should be enhanced, and an early warning system for short-term temperature changes should be established, especially in non-heating seasons, so as to reduce the public health burden caused by temperature change.

### Supplementary Information

**Additional file 1: Fig S1.** The exposure relationship between temperature variability and admissions and deaths for hypertension. Models were adjusted for time trend and seasonal effect, day of week, holiday, mean temperature and relative humidity. Based on Q-AIC, TV07, TV00, TV06 were selected for all O&ER visits, Hospitalization and Deaths.** Fig S2.** The exposure relationship between temperature variability and admissions and deaths for coronary heart disease. Models were adjusted for time trend and seasonal effect, day of week, holiday, mean temperature and relative humidity. Based on Q-AIC, TV04, TV04, TV06 were selected for all O&ER visits, Hospitalization and Deaths.** Fig S3.** The exposure relationship between temperature variability and admissions and deaths for stroke. Models were adjusted for time trend and seasonal effect, day of week, holiday, mean temperature and relative humidity. Based on Q-AIC, TV04, TV07, TV07 were selected for all O&ER visits, Hospitalization and Deaths.** Fig S4.** The percentage change with 95% CI in admissions and deaths for hypertension with 1℃ increase in temperature variability at different exposure days. Models were adjusted for time trend and seasonal effect, day of week, holiday, mean temperature and relative humidity.** Fig S5.** The percentage change with 95% CI in admissions and deaths for coronary heart disease with 1℃ increase in temperature variability at different exposure days. Models were adjusted for time trend and seasonal effect, day of week, holiday, mean temperature and relative humidity.** Fig S6.** The percentage change with 95% CI in admissions and deaths for stroke with 1℃ increase in temperature variability at different exposure days. Models were adjusted for time trend and seasonal effect, day of week, holiday, mean temperature and relative humidity.** Table S1.** The exposure relationship between temperature variability and admissions and deaths for CVDs.** Table S2.** The percentage change with 95% CI in admissions and deaths for CVDs with 1℃ increase in TV at different exposure days.

## Data Availability

The datasets used and/or analyzed during the current study available from the corresponding author on reasonable request.
