# Supplementary material for: A study of temperature variability on admissions and deaths for cardiovascular diseases in Northwestern China
Source: BMC Public Health. 2023 Sep 8;23:1751. doi: 10.1186/s12889-023-16650-3 (PMC10486070; doi:10.1186/s12889-023-16650-3)
Supplement: Supplementary file 1 — Additional file 1: Fig S1. The exposure relationship between temperature variability and admissions and deaths for hypertension. Models were adjusted for time trend and seasonal effect, day of week, holiday, mean temperature and relative humidity. Based on Q-AIC, TV07, TV00, TV06 were selected for all O&ER visits, Hospitalization and Deaths. Fig S2. The exposure relationship between temperature variability and admissions and deaths for coronary heart disease. Models were adjusted for time trend and seasonal effect, day of week, holiday, mean temperature and relative humidity. Based on Q-AIC, TV04, TV04, TV06 were selected for all O&ER visits, Hospitalization and Deaths. Fig S3. The exposure relationship between temperature variability and admissions and deaths for stroke. Models were adjusted for time trend and seasonal effect, day of week, holiday, mean temperature and relative humidity. Based on Q-AIC, TV04, TV07, TV07 were selected for all O&ER visits, Hospitalization and Deaths. Fig S4. The percentage change with 95% CI in admissions and deaths for hypertension with 1℃ increase in temperature variability at different exposure days. Models were adjusted for time trend and seasonal effect, day of week, holiday, mean temperature and relative humidity. Fig S5. The percentage change with 95% CI in admissions and deaths for coronary heart disease with 1℃ increase in temperature variability at different exposure days. Models were adjusted for time trend and seasonal effect, day of week, holiday, mean temperature and relative humidity. Fig S6. The percentage change with 95% CI in admissions and deaths for stroke with 1℃ increase in temperature variability at different exposure days. Models were adjusted for time trend and seasonal effect, day of week, holiday, mean temperature and relative humidity. Table S1. The exposure relationship between temperature variability and admissions and deaths for CVDs. Table S2. The percentage change with 95% CI in admissions and deaths [file 12889_2023_16650_MOESM1_ESM.docx]

**A study of temperature variability on admissions and deaths for cardiovascular diseases in Northwestern China**

**Supplementary material:**


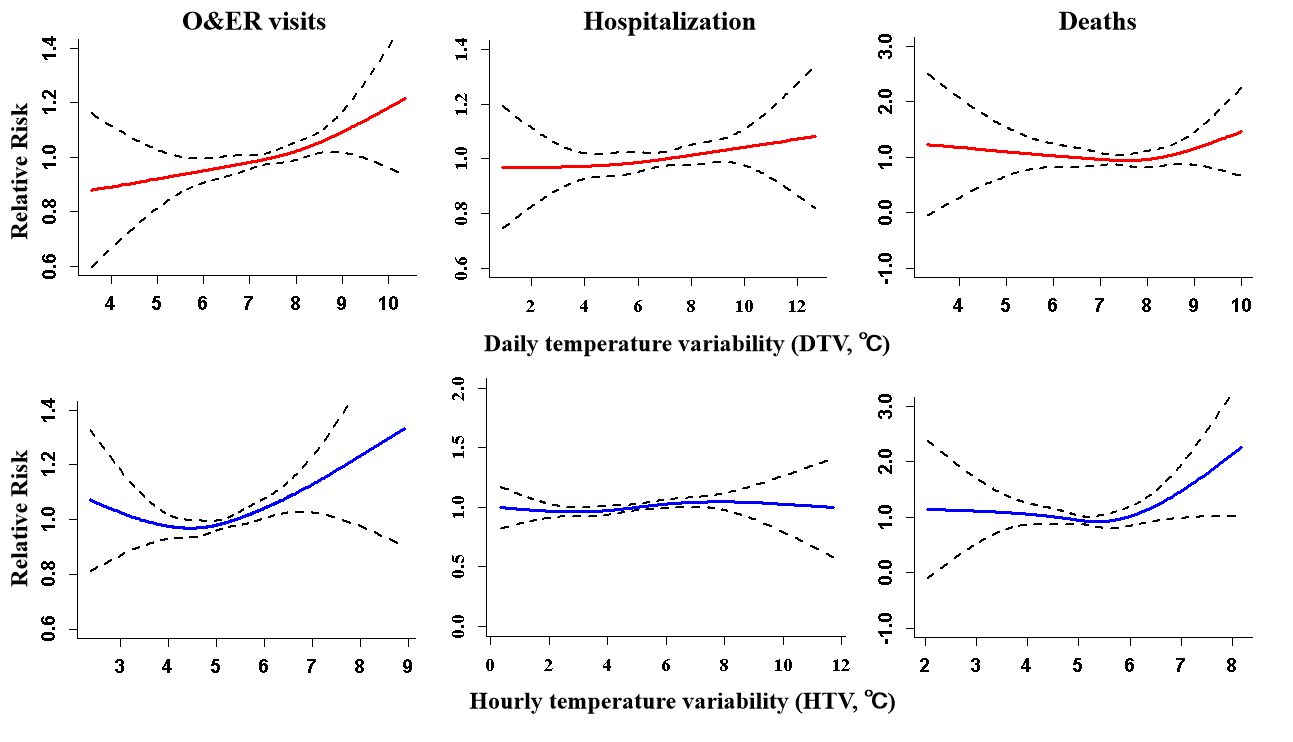


**Fig S1.** The exposure relationship between temperature variability and admissions and deaths for hypertension. Models were adjusted for time trend and seasonal effect, day of week, holiday, mean temperature and relative humidity. Based on Q-AIC, TV07, TV00, TV06 were selected for all O&ER visits, Hospitalization and Deaths.


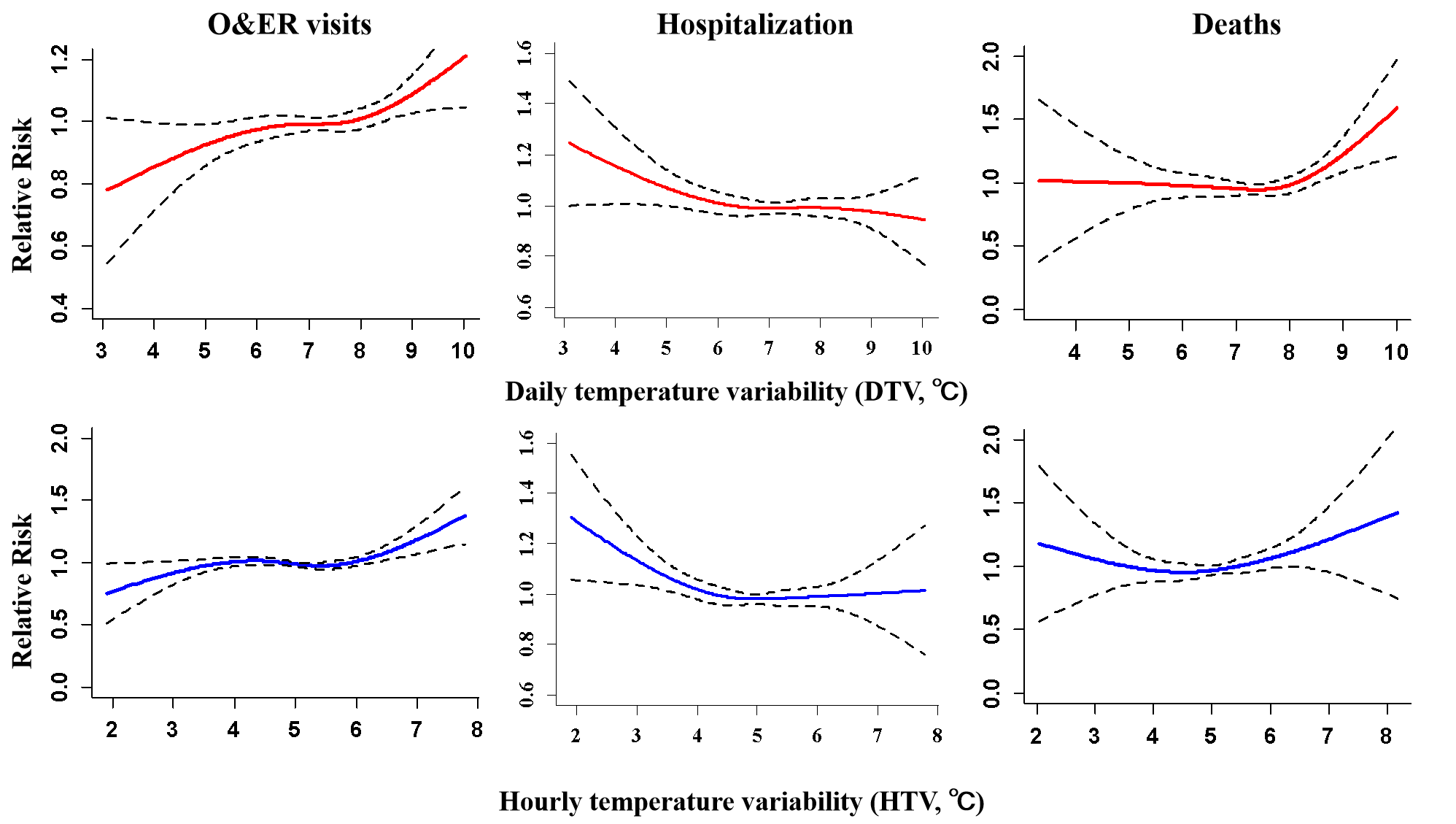


**Fig S2.** The exposure relationship between temperature variability and admissions and deaths for coronary heart disease. Models were adjusted for time trend and seasonal effect, day of week, holiday, mean temperature and relative humidity. Based on Q-AIC, TV04, TV04, TV06 were selected for all O&ER visits, Hospitalization and Deaths.


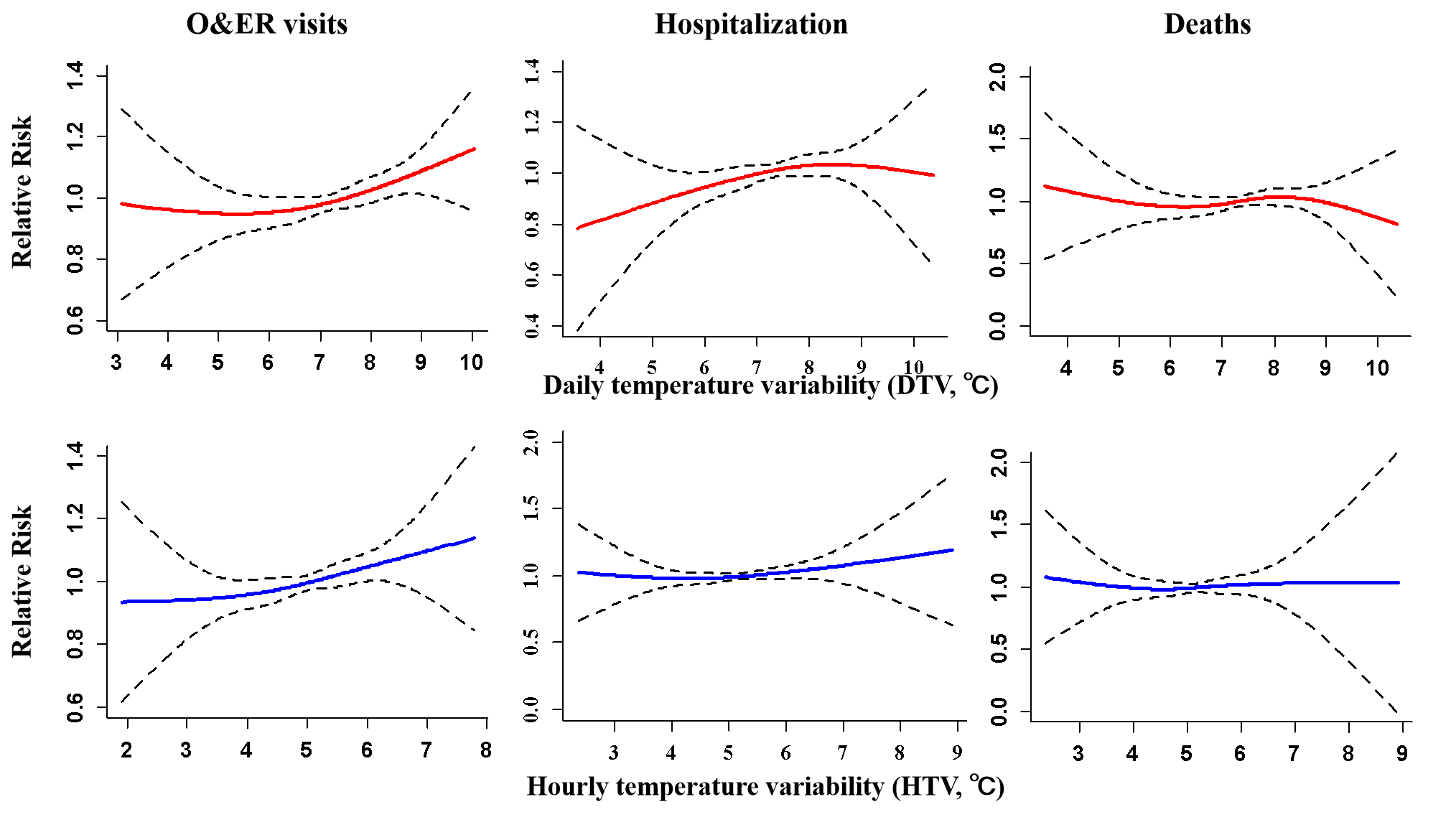


**Fig S3.** The exposure relationship between temperature variability and admissions and deaths for stroke. Models were adjusted for time trend and seasonal effect, day of week, holiday, mean temperature and relative humidity. Based on Q-AIC, TV04, TV07, TV07 were selected for all O&ER visits, Hospitalization and Deaths.


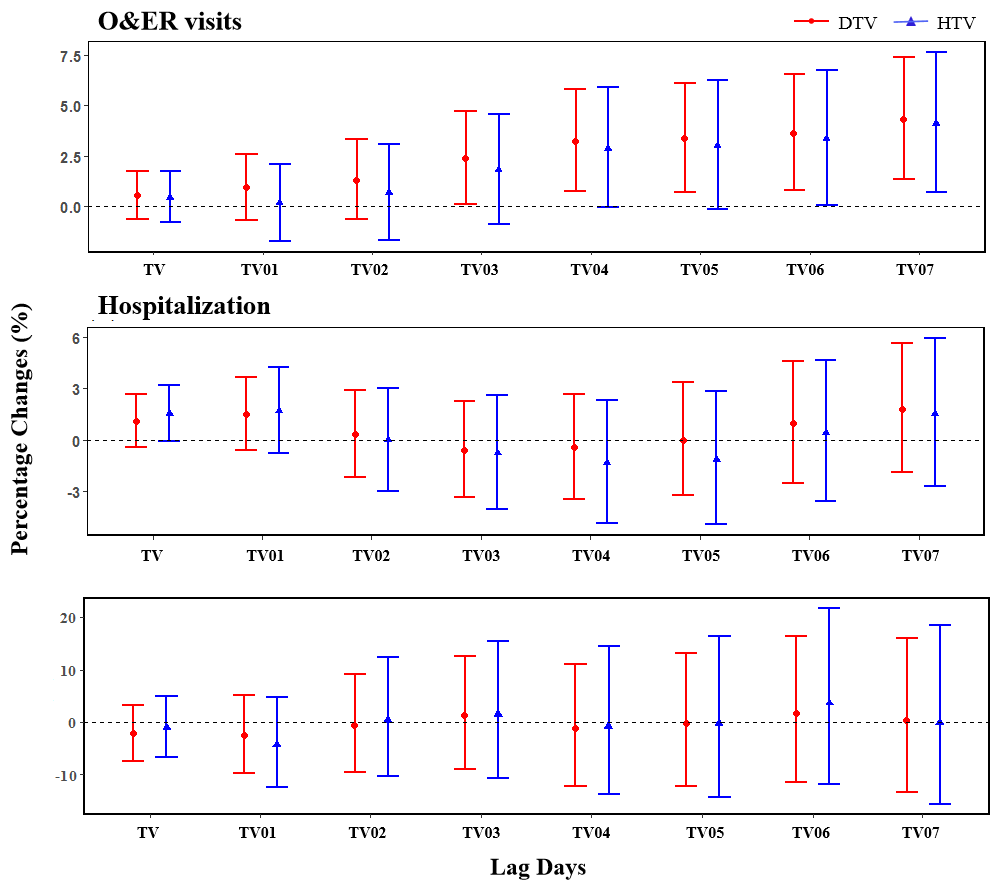


**Fig S4.** The percentage change with 95% CI in admissions and deaths for hypertension with 1℃ increase in temperature variability at different exposure days. Models were adjusted for time trend and seasonal effect, day of week, holiday, mean temperature and relative humidity.

##


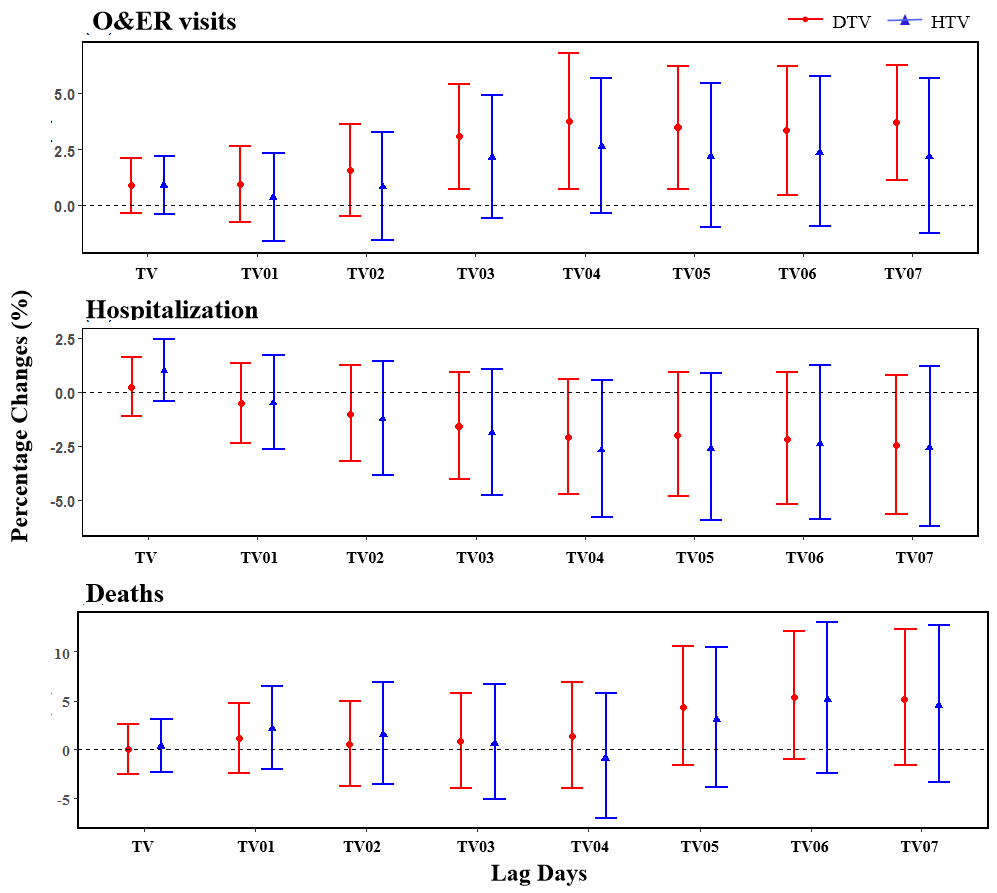


**Fig S5.** The percentage change with 95% CI in admissions and deaths for coronary heart disease with 1℃ increase in temperature variability at different exposure days. Models were adjusted for time trend and seasonal effect, day of week, holiday, mean temperature and relative humidity.


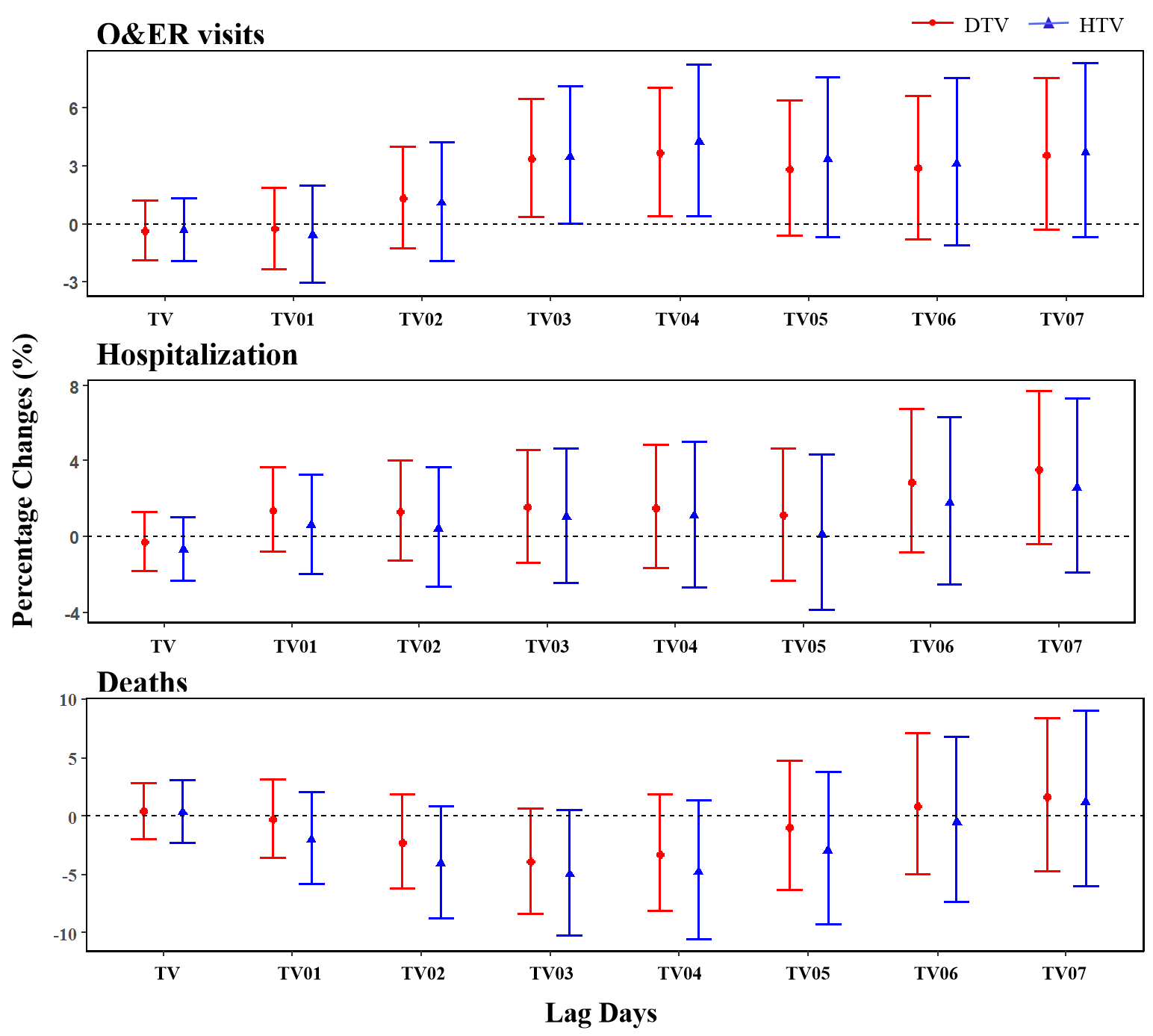


**Fig S6.** The percentage change with 95% CI in admissions and deaths for stroke with 1℃ increase in temperature variability at different exposure days. Models were adjusted for time trend and seasonal effect, day of week, holiday, mean temperature and relative humidity.

**Table S1.** The exposure relationship between temperature variability and admissions and deaths for CVDs.

|  | **DTV (℃)** | | **HTV (℃)** | |
| --- | --- | --- | --- | --- |
|  | <7 | ≥7 | <5 | ≥5 |
| **O&ER visits** |  |  |  |  |
| All cardiovascular disease | \| 0.97 (0.93,1.02) \| \| --- \| | **1.08 (1.03,1.12)** | 0.94 (0.89,1.00) | **1.05 (1.00,1.11)** |
| Hypertension | 0.96 (0.91,1.02) | **1.07 (1.02,1.13)** | 0.93 (0.87,1.00) | **1.06 (1.00,1.12)** |
| CHD | 0.96 (0.90,1.03) | **1.08 (1.02,1.15)** | 0.95 (0.88,1.02) | 1.05 (0.97,1.12) |
| Stroke | 1.02 (0.92,1.13) | **1.09 (1.01,1.17)** | 0.96 (0.86,1.07) | 1.03 (0.94,1.13) |
| **Hospitalization** |  |  |  |  |
| All cardiovascular disease | 1.01 (0.99,1.02) | 1.00 (0.97,1.02) | 1.01 (0.99,1.03) | 0.99 (0.96,1.01) |
| Hypertension | 1.00 (0.97,1.03) | 1.01 (0.97,1.05) | 1.00 (0.96,1.04) | 1.00 (0.96,1.04) |
| CHD | 1.02 (0.99,1.05) | 0.97 (0.94,1.01) | 1.03 (0.99,1.06) | 0.97 (0.94,1.01) |
| Stroke | 0.98 (0.95,1.02) | 1.03 (0.98,1.07) | 0.99 (0.95,1.03) | 1.01 (0.96,1.06) |
| **Deaths** |  |  |  |  |
| All cardiovascular disease | 1.00 (0.90,1.10) | 1.04 (0.97,1.12) | 1.01 (0.91,1.12) | 1.04 (0.94,1.14) |
| Hypertension | 1.00 (0.71,1.40) | **1.33 (1.02,1.73)** | 0.96 (0.67,1.37) | 1.30 (0.95,1.77) |
| CHD | 0.97 (0.83,1.15) | **1.16 (1.03,1.30)** | 1.04 (0.88,1.24) | **1.17 (1.01,1.36)** |
| Stroke | 0.97 (0.83,1.12) | 0.94 (0.84,1.06) | 0.97 (0.82,1.14) | 0.94 (0.81,1.09) |

Bold type indicates *P*< 0.05. Models were adjusted for time trend and seasonal effect, day of week, holiday, mean temperature and relative humidity.

**Table S2.** The percentage change with 95% CI in admissions and deaths for CVDs with 1℃ increase in TV at different exposure days.

|  | **DTV** | | **HTV** | |
| --- | --- | --- | --- | --- |
|  | Lag10 | Lag15 | Lag10 | Lag15 |
| **O&ER visits** |  |  |  |  |
| All cardiovascular disease | **4.47 (1.73,7.28)** | **5.10 (1.97,8.32)** | **3.80 (0.38,7.34)** | **4.95 (1.13,8.92)** |
| Hypertension | **5.61 (2.23,9.11)** | **6.40 (2.56,10.38)** | **5.06 (0.83,9.47)** | **6.64 (1.95,11.55)** |
| CHD | **4.47 (1.07,8.00)** | **5.83 (1.90,9.92)** | 3.74 (-0.53,8.20) | **5.41 (0.58,10.47)** |
| Stroke | 4.41 (0.01,8.99) | 4.68 (-0.37,9.97) | 4.41 (-1.04,10.17) | 4.05 (-2.01,10.48) |
| **Hospitalization** |  |  |  |  |
| All cardiovascular disease | 1.28 (-0.92,3.53) | 1.81 (-0.81,4.50) | 1.27 (-1.56,4.19) | 1.92 (-1.31,5.25) |
| Hypertension | -0.03 (-3.88,3.98) | 1.78 (-2.86,6.65) | -0.01 (-4.90,5.13) | 1.06 (-4.55,7.00) |
| CHD | -2.05 (-5.34,1.35) | -2.20 (-6.09,1.85) | -3.03 (-7.20,1.32) | -2.99 (-7.71,1.97) |
| Stroke | 2.58 (-1.53,6.87) | 1.52 (-3.28,6.55) | 3.20 (-2.07,8.75) | 2.38 (-3.52,8.64) |
| **Deaths** |  |  |  |  |
| All cardiovascular disease | 2.64 (-2.05,7.56) | 2.39 (-2.71,7.77) | -0.16 (-5.87,5.90) | -0.53 (-4.92,4.06) |
| Hypertension | 9.21 (-7.90,29.50) | 8.03 (-10.27,30.06) | 7.58 (-13.26,33.42) | 4.94 (-10.94,23.65) |
| CHD | 4.20 (-3.39,12.39) | 3.91 (-4.35,12.89) | 1.66 (-7.62,11.88) | 0.45 (-6.68,8.12) |
| Stroke | 0.77 (-6.35,8.43) | 1.46 (-6.36,9.92) | -2.22 (-10.91,7.31) | -0.70 (-7.52,6.62) |

Bold type indicates *P*< 0.05. Models were adjusted for time trend and seasonal effect, day of week, holiday, mean temperature and relative humidity.
